# Dr. Frank P. Abbot

**Published:** 1887-02

**Authors:** 


### Obituary.

---

DR. FRANK P. ABBOT.—The many friends of Dr. Abbot received a severe blow in the announcement of his death, which took place in Blasewitz, near Dresden, Germany. He had been a sufferer for many years from an asthmatic trouble, but he bore this, as he bore everything, with so much fortitude that any serious result was not anticipated.

It is very difficult at times to be reconciled to the inevitable,—to feel that the old, old story of death has again to be gone over with its long separations, and the deeper agonies of those nearest and truest; but in this case there is the consolation that his life has been a benediction and his example one worthy of emulation.

It is too true that often the individual must wait the closing of the grave before the finer incentives that have actuated his life can be appreciated. This was not true of the subject of this sketch. He numbered his friends in many lands. In Germany, where he was best known, the time will be long before this life and its deeds will fade from the records of memory. This is the more remarkable when it is considered that Dr. Abbot never thrust himself prominently before the public or his profession. His contributions to dental literature were not extensive, nor did he actively enter into its discussions; but his interest in it was deep and lasting, and he was ever to be found at the front in active work.

He was born in Maine, and graduated at the Baltimore College of Dental Surgery in 1851, leaving shortly afterwards, the same year, for Berlin, thus becoming one of the pioneers of American dentistry in Europe. He drew into his professional life the teachings of Harris, and never in the long course of his extensive practice seemed to lose that influence. While active in interest in every new step that promised advancement, he was slow to adopt changes, preferring the old way. He seemed to the writer to be a remarkable combination of the liberal with a good share of conservative feeling in a professional sense. He was conscientious, and no one could help admiring his strong devotion to principle in refusing to be tempted to use materials which he might regard as objectionable. While he seemed thus to be opposed naturally to innovations, his courage was equal to any change that coincided with his judgment, though it might be in opposition to established rule. This was manifest in his faithful advocacy of tin and gold foils combined in one filling. He disclaimed any originality in this, but accepted it, and it was through his persistent efforts that this has come to be recognized as a valuable addition to practice.

His life, however, is truly his best monument. He was respected and loved by all classes. His advice was sought for by both Americans and Germans. His home was an open one, and his receptions were always enjoyable and crowded occasions. Speaking several languages fluently, he entertained largely many nationalities. The writer was most impressed, in his intimacy with Dr. Abbot, with his power over individuals. There was nothing of the sycophant about him. He was true always to his American instinct, and yet few men can say to the same extent as he could that they were held not only in high estimation, but even enjoyed the affectionate regard of some of the highest in his adopted country. It was always a pleasure to the writer to hear the upper classes of Hanover—the nobility of the old *regime*—speak of Dr. Abbot. He had but to say to them professionally go, and they went. His recommendation was law to them, and by a proper use of this power he was always able to help, provided he was convinced of the worthiness of the subject. His advice was constantly in demand by young practitioners and rarely refused.

A remarkable trait was his ability to accommodate himself to circumstances. It was the privilege of the writer on one occasion to receive from him a lesson in contentment not very soon forgotten. He had suffered great losses financially in stocks after the French and German war of 1870. We were riding together through the environs of Paris. The conversation naturally turned on the beauty of the surroundings and the wealth required to produce these results. The writer ventured to express the deep regret that he felt for Dr. Abbot's serious losses, and that the labor of his lifetime had been so rudely scattered. Responding with a good deal of energy he said, "Don't sympathize with me; you should rather congratulate me, for it was the best thing that could have happened. I can now sleep well at night; have no trouble about stocks, and besides have received a good lesson."

While ever polite in his intercourse with his patients, he seemed to care very little for mere nobility. It is said of him that on one occasion he was called to the Royal palace at the command of the Empress. He waited long, and no Empress appearing, he collected his instruments and departed. This may seem a slight thing to the average American, but it was a very bold thing to do in the presence of royalty. Prince Albrecht, nephew of Emperor William, was always an attached patient of his. This prince held court in Hanover in the palace of the old King George. While operating for the prince on one occasion he asked him, as he laughingly informed the writer, "Why don't you come to me in the car or omnibus, instead of driving here in your carriage?" "That," replied the Prince, "would be a convenient way, but then, you know, I would not be respected."

Dr. Abbot leaves a son to succeed him, who has recently graduated from the dental department of Harvard University. His son-in-law, Prof. Miller, has also been long connected with him, so that the practice he so faithfully labored to perfect is left in competent hands. He married the daughter of Hon. Theodore S. Fay, formerly Minister to Switzerland. His wife and two children survive him. Mr. Fay belonged to that brilliant circle of cultivated men of the past generation who were recognized as a power in the then political world. He resides with his family near Dresden. The daughter of such a man was well fitted to second Dr. Abbot in his social relations, and by her culture and conversational powers added greatly to the comfort of the many who frequented his hospitable home.

Upon the grave of this genial life—this open-hearted friend—the wreath of immortelles may justly be placed. In the presence of the mysteries of creation we are as children; in the presence of death we question the possibilities of an untried path. We linger on the memory of the good and true, and in this, the holiest of temples, we enshrine our friend.—JAMES TRUMAN, *in Dental Cosmos.*

